# Extracting Teeth

**Published:** 1866-12

**Authors:** P. C. Branch

**Affiliations:** Vinton, Iowa


### EXTRACTING TEETH.

BY P. C. BRANCH, VINTON, IOWA.

Read before the Iowa State Dental Society, July, 1868.

VERY little need be said of the deciduous teeth, experience having settled the question, and decided against extracting them at any time before their successors make their appearance, when any fragments remaining unabsorbed may be too easily removed to demand any description of the operation.

It has been said, "to cook a rabbit, first *catch* him;" so, to extract a tooth, first find a tooth so hopelessly diseased as to be incurable and intolerable. When you find one, or a dozen such teeth in a patient's mouth, each one, and all of them, demand immediately, to be extracted.

If an examination reveals one or more exposed and inflamed pulps, or in any condition not absolutely hopeless, while the adjoining and antagonizing teeth are, for the greater part, in a good, or salvable condition, it is evidently *not* the *dentist's* duty to extract. The root, or roots of a bicuspid or molar, if firm in their sockets, and giving no trouble to the wearer, may be wisely let alone, unless an artificial substitute is to be inserted, or the operator is in want of a fee.

The gradual absorption of such roots, and the filling up of the sockets, keeping pace with each other, produce considerably less wasting of the alveolar ridge, and loss of support, to remaining teeth, than is sure to follow their sudden and violent removal.

A condition of acute, and increasing periosteal inflammation, seems unfavorable to extracting; which frequently fails to give relief, and sometimes appears to aggravate the symptoms, and increase the patient's sufferings.

A case of this kind, under the care of a physician, came under the writer's observation. The physician removed the offending tooth, and, at the same time some necrosed roots The operation was followed by an immediate increase and extension of the inflammation, and resulted in the destruction and exfoliation of nearly one half of the lower jaw, and, of course, considerable deformity of the face. The writer would generally defer the operation, until resolution, or suppuration, assures him that the crisis is past. Then the parts are certain to heal kindly.

The practice of removing sound, or salvable teeth, to give place to artificial ones, because there may be but few, or because they are irregular—homely—and *pretty teeth* are desired, cannot be too strongly condemned.

The true dentist, is a *true* artist; and a true artist, venerates Nature, only less than he does the God of Nature. He sees fitness and beauty in her handiwork, and only when *disturbing* forces have *distorted* what *would* have been beautiful, will he dare substitute the productions of art?

If but one sound natural tooth remains in its natural position firmly, the gums adhering well to its neck, or, if there be several such, scattered about the mouth, one in a place, they ought not to be extracted. The resources of dental skill, are, or ought to be, equal to their preservation, and the supply of what has been lost. It is true that the pressure of a plate has a *tendency* to produce absorption of their sockets and gums; but if proper care be exercised to prevent the plate from touching the natural teeth, and, if the bearings be made lightest in their immediate vicinity, that dreaded result will be quite remote, if indeed it comes at all. Besides, the time is gone by for us to talk about making *permanent* sets of teeth. Let our patrons be made to see that the durability of our work depends almost as much upon their care, as upon our skill and faithfulness; and *permanency*, will be more nearly secured than in any other way.

Not unfrequently a man sits down in our chairs, saying, "I want you to pull a tooth for me." An examination satisfies the operator that it may and ought to be saved. But argument, explanation and proof are lost upon him. He has come to have a tooth pulled, and he will not be "humbugged"

into anything else. Justice to a numbscull may urge a compliance with his demand; but a higher allegiance to science and art, requires us to refuse obedience to the dictation of a block-head, and to send him where ignorance and cupidity have fashioned already the " tooth-puller " for such as he.

Humanity should stimulate us to do all within our power to alleviate the sufferings of the poor, but intelligent patient, and suggests, that resort to extracting should not be had, until the organ can no longer be tolerated. Such a conservative course often gives the patient an opportunity, under more prosperous circumstances, to save what would, otherwise, have been irrecoverably lost, and always serves to impress the minds of that very worthy class of people, with the value and importance of their teeth, and our profession.

There remain to be considered the mode of extracting, including, perhaps, the instruments to be used.

A hasty retrospect of twenty years practice strongly inclines the writer to lay down the pen, and abandon his task in despair; for how shall one describe operations almost infinite in variety, and, after long experience, still presenting new difficulties to be surmounted?

The instruments are lancets, forceps, and elevators. The lancet should be of three patterns: No. 1, nearly straight; No. 2, curves about half of one inch from its point, so as to cut, partly behind an adjoining tooth, and by the side of the one to be removed; No. 3, should be bent at a right angle with, and made to cut *transversely* to the line formed by its shank and handle.

This last is used to sever the gum from the saucer-shaped depression, upon the approximal surfaces of the molars. At that point, the gum is capable of resisting more force than can be safely applied, for the removal of a tooth whose crown is greatly weakened by decay; and, the thorough use of this instrument will frequently save operator and patient the mortification and misery of a failure.

The roots of the ten anterior teeth are often held very firmly in their sockets, while the hold for their removal is extremely slight. Here lancet No. 1, comes right to the hand. After loosening the gum, force its point between the root and the alveolars, two to four times, at as many, and *opposite* sides of the former, and the latter will be so loosened from its bony attachments as to be comparatively easily removed.

The gum that overlies and contracts, upon the edges of a root, should be severed by a slit on the labial or buccal side, in the direction of its apex, as far as an instrument is to be introduced for its removal. If forceps are to be used, it may be necessary to make a similar slit on the lingual side, and when it becomes necessary to grasp and cut through the alveolus, there should be short transverse cuts, at the extremeties of those just described. The little flaps thus formed being separated from the alveolus, and sufficient room secured for getting a firm hold, no great difficulty will be met in completing the operation.

While it is true that a great many teeth should be extracted without the lancet, it is equally true that in large numbers of cases it performs a humane, and almost indispensable work.

It is absolutely necessary that the beaks of forceps be well fitted to the teeth they are to extract, strong enough, and so tempered as not to spring or break, with the handles fitting the right hand, and arranged so that the operator can control the patient's head with his left arm and hand. But all unnecessary twists and crooks may be wisely dispensed with.

The elevators are invaluable for extracting roots. The writer uses these forms: No. 1, is simply a strong octagon handled steel excavator, straight, with a slight sharp "hoe-point," and is used to extract the upper anterior roots. The instrument is passed between the gums and root, with the "hoe" edge toward the latter, the handle grasped firmly with the fingers and palm of the hand, the end of the flexed thumb resting against the cutting edge of an adjoining tooth, or the alveolus. When pressed against the root, the "hoe" cuts its own hold, and the extension of the thumb, and the contraction of the fingers, brings away the root.

No. 2, differs from No. 1, only in its point, which is stronger and slightly curved. When held in the right hand, with the thumb extended to the left, the cut will be downward, and toward the operator. Like No. 1, it cuts its own hold, (it may be used upon almost any root in the mouth,) and the root is removed entirely by the contraction of the hand, with the thumb resting upon some safe point in the patient's mouth.

No. 3, is an enlargement and modification of No. 1. Its hoe edge may be slightly serrated to prevent slipping, and bent at a right angle with the shank, one fourth of an inch from the edge.

It may be used to advantage upon the roots of molars and bicuspids. The hold is secured upon the lingual side, the flexed thumb rests against the buceal side of an adjoining tooth, and the root is lifted and rolled out toward the cheek, entirely by *hand* power.

An important condition to success is that the patient's head, and (when that is the seat of the operation) the lower jaw, be held as nearly immovable as possible, for it is plain that every motion produces a loss, if not a dangerous misdirection of force.

After all that has been or might be said, it seems impossible to reduce extracting teeth to a "system." It is far more like an unceasing resort to expedients, reluctantly resorted to by practitioner and patient.

From so disagreeable a duty let us pray, "Good Lord deliver us."

